# Supplementary material for: Understanding of Active Sites and Interconversion of Pd and PdO during CH4 Oxidation
Source: Molecules. 2023 Feb 18;28(4):1957. doi: 10.3390/molecules28041957 (PMC9959627; doi:10.3390/molecules28041957)
Supplement: Supplementary file 1 [file molecules-28-01957-s001.zip › molecules-2161825-supplementary.pdf]

Understanding of Active Sites and Interconversion of Pd and PdO during CH_4 Oxidation

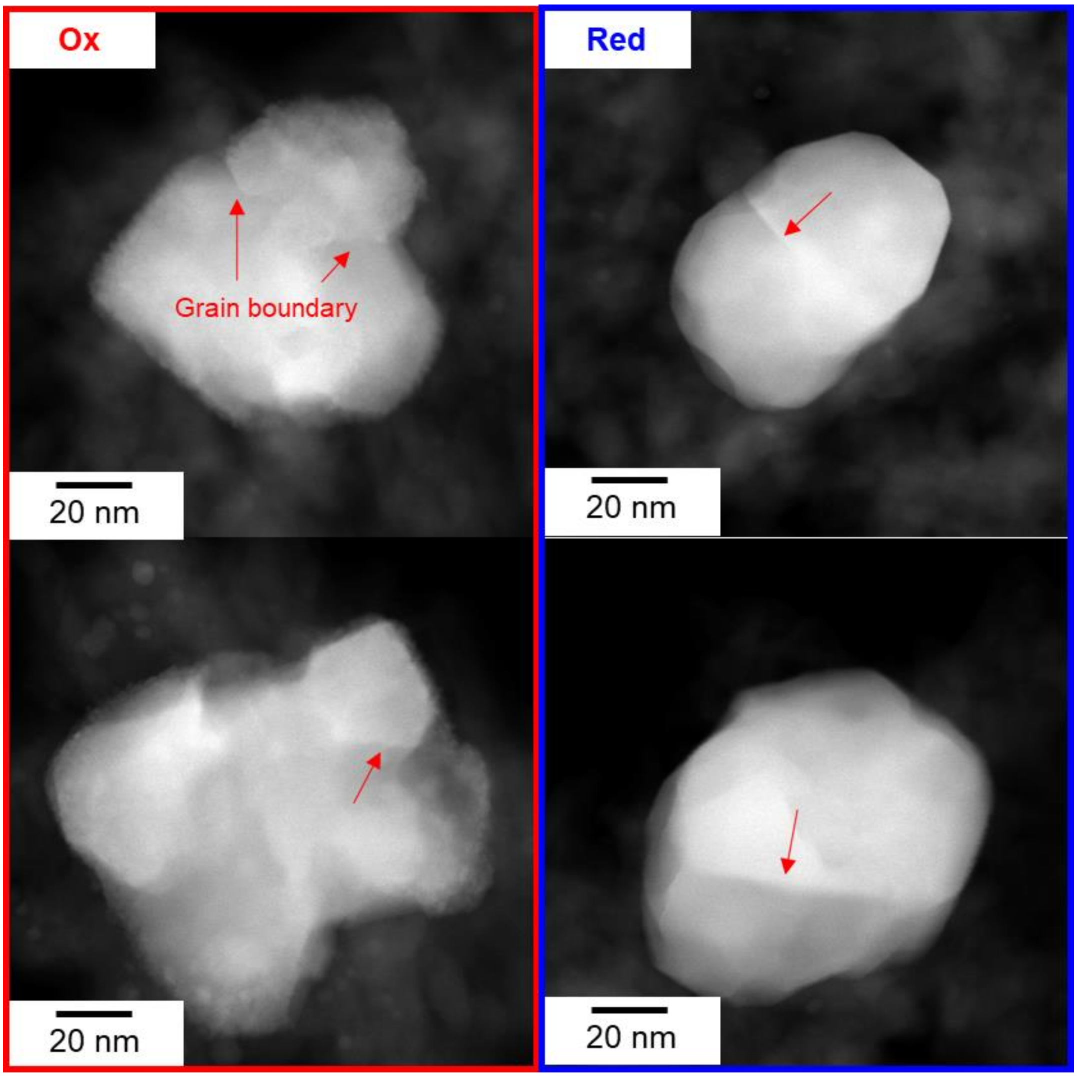

Figure S1. High magnification STEM images of Pd/Al_2O_3-Ox (red) and PdAl_2O_3-Red (blue).

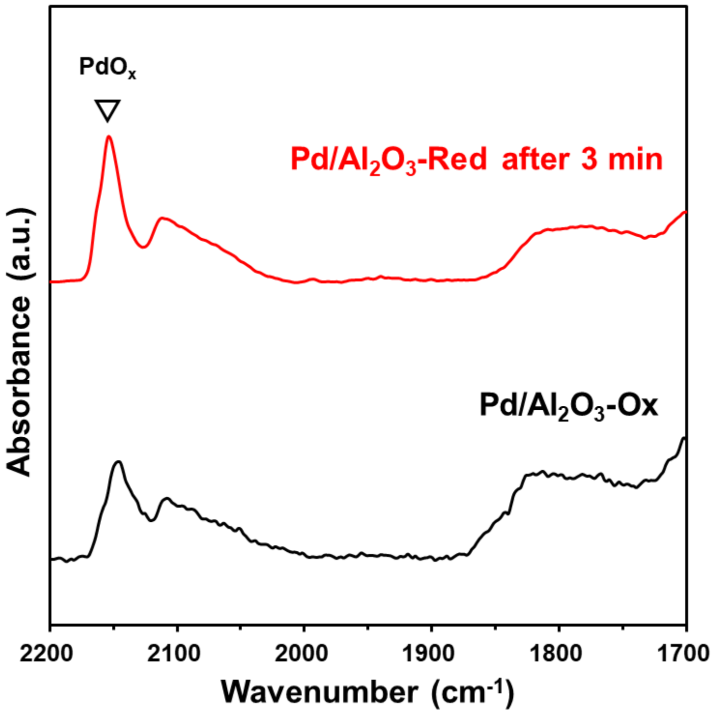

Figure S2. In-situ DRIFTS spectra of adsorbed CO on Pd/Al_2O_3-Ox (black) and Pd/Al_2O_3-Red after 3 min of CH_4 oxidation. DRIFTS spectra were recorded at 25 °C after He purging. The surface reconstruction from Pd to PdO immediately occurred within 3 min of the reaction. CH_4 oxidation condition – 1% CH_4, 10% O_2 in He at 340 °C.
